# Structure Elucidation of Unknown Metabolites in Metabolomics by Combined NMR and MS/MS Prediction

**DOI:** 10.3390/metabo8010008

**Published:** 2018-01-17

**Authors:** Rene M. Boiteau, David W. Hoyt, Carrie D. Nicora, Hannah A. Kinmonth-Schultz, Joy K. Ward, Kerem Bingol

**Affiliations:** 1 Environmental Molecular Sciences Laboratory, Pacific Northwest National Laboratory, Richland, WA 99354, USA; rene.boiteau@pnnl.gov (R.M.B.); david.hoyt@pnnl.gov (D.W.H.); carrie.nicora@pnnl.gov (C.D.N.); 2 Department of Ecology and Evolutionary Biology, University of Kansas, Lawrence, KS 66045, USA; hkinmonth@ku.edu (H.A.K.-S.); joyward@ku.edu (J.K.W.)

**Keywords:** metabolomics, metabolite identification, hybrid MS/NMR method, in silico fragmentation, chemical shift prediction, *Arabidopsis thaliana* metabolome

## Abstract

We introduce a cheminformatics approach that combines highly selective and orthogonal structure elucidation parameters; accurate mass, MS/MS (MS^2^), and NMR into a single analysis platform to accurately identify unknown metabolites in untargeted studies. The approach starts with an unknown LC-MS feature, and then combines the experimental MS/MS and NMR information of the unknown to effectively filter out the false positive candidate structures based on their predicted MS/MS and NMR spectra. We demonstrate the approach on a model mixture, and then we identify an uncatalogued secondary metabolite in *Arabidopsis thaliana*. The NMR/MS^2^ approach is well suited to the discovery of new metabolites in plant extracts, microbes, soils, dissolved organic matter, food extracts, biofuels, and biomedical samples, facilitating the identification of metabolites that are not present in experimental NMR and MS metabolomics databases.

## 1. Introduction

Metabolomics has become a key discipline in small molecule research. It has been widely applied to generate new hypotheses for unresolved scientific questions, such as in metabolism-related disorders, environmental carbon cycling studies, complex biofuel analysis, nutritional and food sciences, and synthetic biology optimization [1,2,3,4,5]. Nuclear magnetic resonance (NMR) spectroscopy and mass spectrometry (MS) are the two most powerful experimental methods for metabolomics [6,7]. This is primarily because of their high-resolution powers providing detection of individual molecular species in a short acquisition time with minimal sample preparation. Connecting the detected signals to scientific interpretation requires assignment of individual signals to their corresponding metabolite identities. This is often performed by querying detected signals against one or several experimental databases such as BMRB [8], HMDB [9], METLIN [10] and COLMAR [11]. The success of this approach for positive metabolite identification requires the presence of the spectra of a metabolite within the database. Although excellent progress has been made in growing the number of metabolites in databases, many of the signals detected in metabolomics experiments cannot be identified through this database approach because of the absence of their spectra in metabolomics databases.

Identification of these unknowns is a major bottleneck. Traditionally, identification of unknowns requires extensive isolation of the molecule from complex mixture in sufficient amount for detailed analysis by NMR and other spectroscopic techniques [12]. While this approach has been proven useful, complete fractionation is time-consuming. Moreover, low yield of purification may not allow downstream structure elucidation processes for low-abundance metabolites. Alternatively, structure elucidation can be performed in the mixture environment, such as in crude extract or partially fractionated sample. This strategy is highly attractive for high-throughput metabolomics. There are three main approaches proposed for this purpose. The first approach excludes spectroscopic analysis; rather, it aims to identify metabolites by LC-MS and LC-MS/MS techniques. This approach compares experimental MS/MS (MS^2^) spectra of an unknown metabolite with the predicted MS/MS fragmentation of possible candidate structures to find the best match. This approach has received significant attention in recent years. There are multiple publicly available software and web servers allowing the query of experimental LC-MS and LC-MS/MS data for identification of unknowns, including MetFrag and CSI:FingerID [13,14,15].

The second strategy, on the other hand, relies on the NMR technique, in which the experimental chemical shifts of unknown metabolites are sequentially assigned and deconvoluted by multidimensional NMR [16,17]. These assignments are further verified through comparison with their quantum NMR chemical shift predictions [18]. While these MS- and NMR-based approaches greatly facilitate the structural characterization, they have limited power, since they rely only on a single technique. Recently, a hybrid MS/NMR metabolite identification strategy, SUMMIT MS/NMR has been proposed. SUMMIT first acquires experimental high-resolution MS^1^ and NMR spectra of a metabolomics sample. Then, it compares experimental NMR chemical shifts of the unknowns in the sample with the predicted NMR chemical shifts of all feasible structures consistent with the derived chemical formulas from high resolution MS^1^ [19]. Recently, this approach was extended to 3D NMR and FT-ICR mass spectrometry to increase resolution and accuracy of metabolite identification [20].

Here, we will further explore improving the accuracy of NMR/MS-based approaches for structure elucidation by combining NMR predictions with MS/MS predictions from a public in silico MS/MS library. This is the first cheminformatics approach that compares and combines MS/MS and NMR predictions for unknown/uncatalogued metabolite identification. We demonstrate the approach on a model mixture and then we identify an uncatalogued secondary metabolite in *Arabidopsis thaliana*.

## 2. Results

### 2.1. Identification of a Known Metabolite without the Use of a Database for Matching

**Figure 1:**
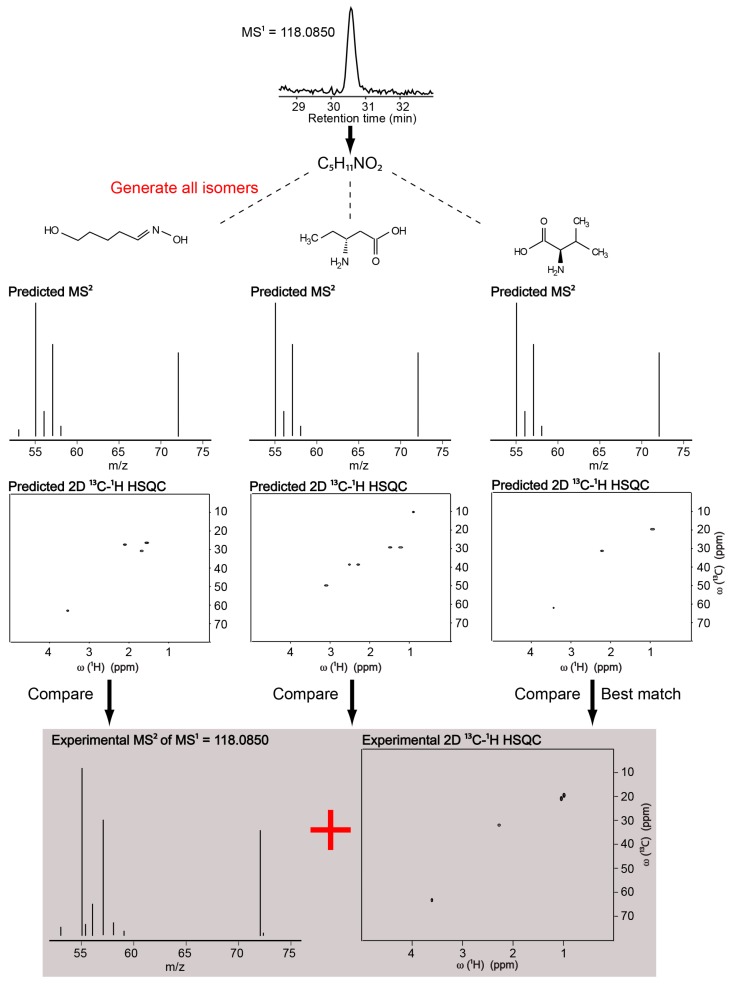
Workflow of the NMR/MS^2^ approach on a single metabolite (valine) in the NMR spectrum.

Our NMR/MS-based approach works as follows: we first determine the chemical formula of an unknown LC-MS feature by analyzing its accurate mass and isotopic distributions. Next, we generate all possible candidate structures consisted with the chemical formula. We predict the MS/MS and NMR spectra of each candidate structure. Meanwhile, we collect the experimental NMR spectrum of the same sample. We compare the predicted MS/MS and NMR data against the experimental MS/MS and NMR, and rank them according to the level of agreement to determine the best matching candidate. Figure 1 shows the approach on a simplest case, where there is only one metabolite in the NMR spectrum; therefore, NMR spectrum was paired with the LC-MS spectrum. We used the MS^2^ and NMR spectra of valine. The high-resolution LC-MS spectrum of valine allowed the determination of its molecular formula, C_5_H_11_NO_2_. All possible structures consistent with C_5_H_11_NO_2_ were determined using the ChemSpider database [21]. There were 453 possible structures. We queried all the 453 MS^2^ predictions against the experimental MS^2^ of valine by using the MetFrag web server [15]. This gave a scoring for each predicted MS^2^ match. Valine MS^2^ prediction was the 6th-best match to the experimental MS^2^ of valine among 453 predictions. We predicted the 2D ^13^C-^1^H HSQC NMR spectra [22] of all the 453 structures by using MestReNova software, and queried them against the experimental 2D ^13^C-^1^H HSQC of valine. Valine NMR prediction was the best match to the experimental NMR spectrum of valine.

By using the same strategy, we analyzed nine other common metabolites. Table 1 (column 7) and Table 2 (column 5) show the performance of MS^2^ and NMR predictions for these metabolites, respectively. With one exception, the NMR predictions were always either the best or second-best match to their experimental signals; the NMR prediction of panthothenate was the fourth-best match. On the MS^2^ side, the predictions matched less successfully to their corresponding experimental MS^2^ (Table S1). For instance, nicotinate and glutamine predictions were the 7th- and 10th-best matches, respectively. Phenylalanine was an exception, with the MS^2^ prediction being the best match to its experimental data.

Overall, NMR predictions turned out to be a more effective filter than MS^2^ predictions. Thus, the structural assignments obtained with NMR prediction tend to provide the most accurate structural information, with MS^2^-based predictions providing an orthogonal method that can distinguish between molecules that yield similar NMR chemical shifts. For instance, proline NMR prediction was the second best to its experimental NMR spectrum with a chemical shift difference of 0.3077 and 1.0908 ppm along ^1^H and ^13^C dimension. The best match was 5-hydroxy-2-piperidinone with a chemical shift difference of 0.1072 and 2.1928 ppm along ^1^H and ^13^C dimension. Whereas MS^2^ ranks of proline and 5-hydroxy-2-piperidinone were 26 (score: 0.8515) and 167 (score: 0.5964), respectively (Figure S1). Therefore, the false positive candidate (5-hydroxy-2-piperidinone) based on NMR prediction can be eliminated by MS^2^ prediction, showing the advantage of combining these two orthogonal parameters for metabolite identification.

### 2.2. Identification of Known Metabolites in a Test Mixture without the Use of a Database for Matching

In the example above, which considered one unknown metabolite at a time, each NMR spectrum was paired with a single LC-MS feature. However, it is common in metabolomics to analyze complex mixtures consisting of multiple metabolites within unfractionated samples. In these cases, multiple LC-MS features and multiple deconvoluted NMR spectra are generated, without knowing which pairs correspond to the same metabolite. This increases the challenge of metabolite identification. The NMR/MS^2^ approach can also be applied to mixtures containing multiple metabolites. When multiple metabolites are present, the workflow is modified to consider all pairwise combinations; NMR predictions are generated for all structural isomers of each LC-MS feature, and they are all compared to every deconvoluted experimental NMR spectra.

**Figure 2:**
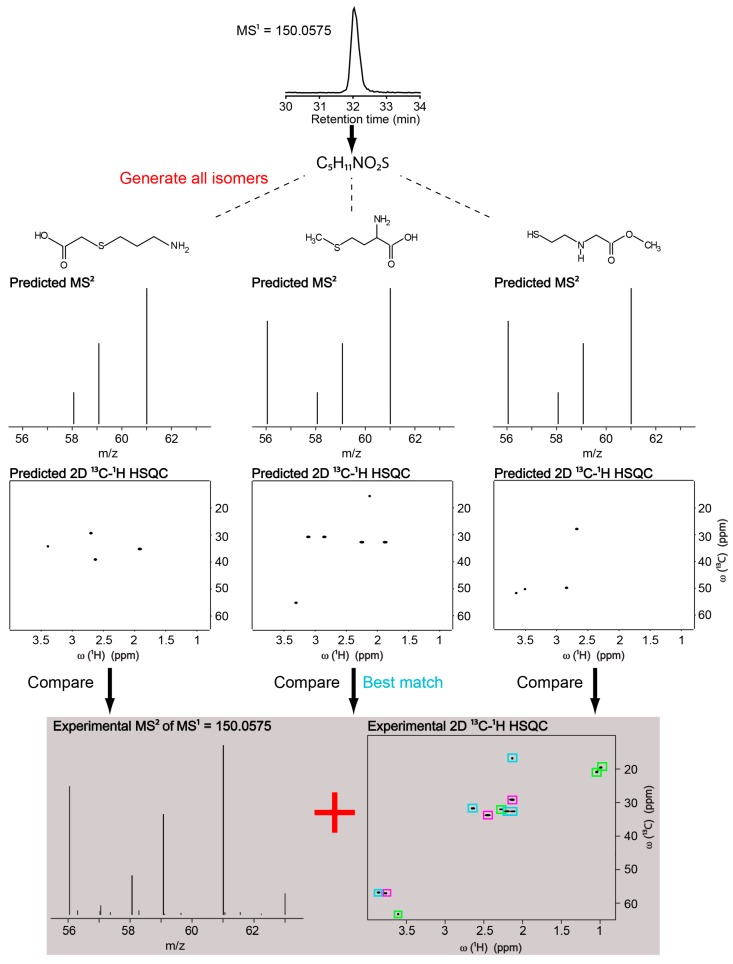
Identification of methionine by the NMR/MS^2^ approach in a three-metabolite mixture. Magenta, light-blue and green boxes indicate HSQC peaks of glutamine, methionine and valine, respectively.

Figure 2 shows the approach on a sample consisting of three metabolites; valine, methionine and glutamine. Deconvolution of the HSQC spectrum by connectivity information from TOCSY [23] and HMBC [24] resulted in 3 HSQC-peak lists, colored magenta, light-blue and green (Figure 2). The LC-MS feature of methionine was first converted to the chemical formula C_5_H_11_NO_2_S. There were 212 possible isomers. We queried HSQC predictions of the 212 structures against the HSQC peak lists of the magenta, light-blue and green individually. Among them, the best match was the one between the predicted HSQC of methionine and the light-blue peaks, corresponding to the experimental HSQC of methionine (Figure 2). The predicted HSQC of methionine provided a better match to the light-blue peaks than all the other 211 methionine isomers compared to all three deconvoluted peak lists.

To demonstrate how the approach would work in a more complex mixture, we expanded the list of metabolites from three to ten; thymidine, proline, phenylalanine, pantothenate, nicotinate, methionine, isoleucine, glutamine, leucine and valine. Table 2, column 6 shows the performance of NMR prediction in the ten-metabolite mixture. The ranks of thymidine, proline, phenylalanine, nicotinate, methionine, isoleucine, glutamine and valine did not differ from being the only metabolite in the NMR spectrum (column 5) to being one of the ten metabolites in the mixture (column 6). On the other hand, the rank of panthothenate and leucine increased from 4 to 16 and from 1 to 3, respectively. This is because in the single-metabolite case, NMR predictions of panthothenate and leucine isomers were limited to matching only to the experimental NMR spectra of panthothenate and leucine. Whereas in the mixture, they can also match to experimental NMR signals of the other mixture metabolites. Figure S2 shows this in detail for leucine. When NMR predictions of 962 C_6_H_13_NO_2_ isomers (including leucine and isoleucine) were queried against the experimental HSQC peak-lists of the ten metabolites, the best-matching NMR prediction belonged to isoleucine, which found the experimental NMR of isoleucine as the best match (true positive). The second-best match was 3-methylaminopropyl acetate, which found experimental NMR of proline as a match (false positive). Finally, the third-best match was between the predicted NMR of leucine and the experimental NMR of leucine (true positive).

3-methylaminopropyl acetate can be eliminated by MS^2^ prediction, since MS^2^ prediction of 3-methylaminopropyl acetate was ranked 92nd (score: 0.6665), whereas MS^2^ prediction of isoleucine was ranked 22nd (score: 0.8723) when they were compared to experimentally detected MS^2^ of isoleucine. MS^2^ prediction of 3-methylaminopropyl acetate was ranked 585th (score: 0.2079), whereas MS^2^ prediction of leucine was ranked 148th (score: 0.7002) when they were compared to experimentally detected MS^2^ of leucine. Therefore, this shows, again, the advantage of combining NMR and MS^2^ predictions for metabolite identification.

### 2.3. Identification of Uncatalogued Metabolite in Arabidopsis thaliana

**Figure 3:**
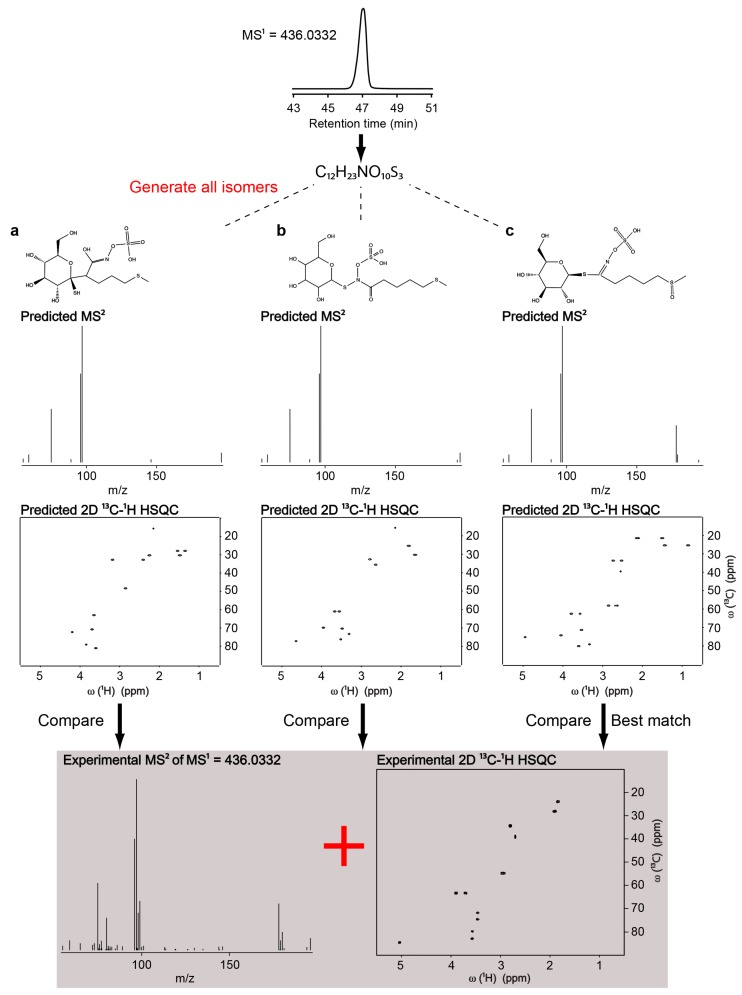
Identification of glucoraphanin in *Arabidopsis* extract by using the NMR/MS^2^ approach.

Many of the metabolites in plants are unknown or uncatalogued; therefore, plants are excellent samples to demonstrate the power of the NMR/MS^2^ approach. We recorded the LC-MS and LC-MS/MS spectra of *Arabidopsis* extract and queried this data against the METLIN database [10], but could not identify many of the high-abundance peaks. Similarly, we recorded the 2D ^13^C-^1^H HSQC spectrum of the sample, and queried this against COLMAR HSQC database [25]. Again, many of the high-abundance peaks could not be identified, as these metabolites were not in either database. One of the highly abundant metabolites had an *m*/*z* 436.0332 in negative mode at the retention time ~47 min (Figure S3). The challenge of identifying unknown metabolites starts at the chemical formula determination step. Previous studies have shown that even 1 ppm mass accuracy is often insufficient to unambiguously determine the chemical formula without additional constraints from isotopic patterns [26]. For the accurate mass 436.0332, there are 4291 possible structures in ChemSpider within 30 ppm mass error, 3194 possible structures within 20 ppm mass error, 1909 structures within 10 ppm mass error, 907 structures within 5 ppm mass error, 528 structures within 3 ppm mass error and 379 structures within 1 ppm mass error. By including the isotopic distribution, we narrowed down the possible chemical formula to C_12_H_23_NO_10_S_3_. The next challenge was to determine which of the many isomers of that formula match the structure of the unknown molecule. We queried all MS^2^ predictions of all possible isomers of C_12_H_23_NO_10_S_3_ against the experimental MS^2^ by using the MetFrag web server. MetFrag hits with their matching scores are shown in Figure 3. The structure **c** was the best match with a matching score of 1.00, structure **b** was the second-best match (score of 0.87), and structure **a** was the third-best match (score of 0.79). The main MS/MS peak differentiating **c** from **a** and **b** was at *m*/*z* 178.0166 (Figure 3), which corresponds to the theoretical mass of the fragment formula of [C_5_H_9_NO_4_S]-H^−^, that includes the SO_4_ group and part of the aliphatic chain (Figure S4).

For NMR, we simplified the plant extract into sub-mixtures by performing LC-fractionation at 1 min intervals. At the retention time ~47 min, we obtained the 2D ^13^C-^1^H HSQC spectrum in Figure S5. Querying the HSQC predictions of all possible isomers against the experimental HSQC (Figure 3) also returned the structure **c** as the best match with average chemical shift ppm error of 0.2081 ppm in ^1^H and 1.4187 ppm in ^13^C dimension. The key contributor for the differentiation of molecule **c** from **a** and **b** was the methyl group at the end of the aliphatic chain. In molecule **c,** the methyl was attached to a sulfoxide group, while in molecule **a** and **b** it was attached to a thioether. The prediction values of the methyl group in structure **c** was 2.55 ppm in ^1^H and 39.57 ppm in ^13^C dimension, whereas the prediction values of the methyl group in structure **a** and **b** were both 2.15 ppm in ^1^H and 15.64 ppm in ^13^C dimension. It was experimentally detected in 2.70 ppm in ^1^H and 39.06 ppm in ^13^C dimension (Figure 3). For final validation, we purchased metabolite **c** (glucoraphanin) and recorded the HSQC spectrum. Close agreement between the uncatalogued molecule from *Arabidopsis* and the purchased standard confirmed the identity (Figure S6). The NMR/MS^2^ approach successfully identified glucoraphanin in *Arabidopsis*.

## 3. Discussion

The introduced NMR/MS^2^ approach is highly compelling for identification of unknown and uncatalogued metabolites for multiple reasons. First, it combines highly selective, universal and orthogonal structure elucidation parameters—accurate mass, MS^2^ and NMR—in a single analysis platform to accurately identify metabolites. Secondly, the approach does not require any experimental NMR or MS metabolomics database for identification. Instead, it generates in silico NMR and MS^2^ spectra for all possible isomers of a chemical formula and queries them directly against experimental NMR and MS^2^ spectra of the unknown of interest. As a proof-of-principle, we used the ChemSpider database [21] as a structure generator, assuming it to contain a sizeable portion of the possible isomers of a chemical formula. To expand the structural space, one could replace ChemSpider with a structure generator such as MOLGEN [27], or one could use a more biological structure resource such as KEGG [28] to focus on biological compounds. Third, the introduced approach has a robust workflow. It not only works in single metabolite samples but also in mixtures.

This cheminformatics approach provided a unique platform to compare performances of NMR and MS/MS predictions for structure elucidation. NMR predictions turned out to be a more effective filter than MS/MS predictions in the model mixture study. Overall, the complementarity of NMR and MS/MS predictions increased the accuracy of metabolite identification as compared to using individual techniques alone. We only used a single collision energy (30 V) for fragmentation, perhaps using multiple collision energies or optimizing collision energies for each metabolite and comparing them with other in silico MS/MS tools such as combined energy competitive fragmentation modeling (CFM) [29] and CSI:FingerID [14] may provide better results, this needs to be further tested. To improve the selectivity on the MS-side, one could include additional parameters such as collusion cross-sections from ion mobility mass spectrometry [30] and LC-retention time, although the latter one is highly dependent on experimental conditions. Determination of the correct molecular formula or at least a small set of possible formulas including the correct formula is crucial for downstream analysis. Accurate mass significantly lowers the number of possible structures that are consistent with the detected mass. For cases where accurate mass is still not enough to narrow down to a single formula, the NMR/MS^2^ approach still works, as the combined MS^2^ and NMR predictions are strong enough to filter majority of false positive structures coming from false chemical formulas. Higher resolution MS systems than TOF, such as Orbitrap and FTICR, would provide better performance, especially for larger metabolites.

With NMR, we used 2D ^13^C-^1^H HSQC [22] predictions as an effective filter, predictions were performed by empirical calculations. The advantage of empirical calculations is the speed. Each prediction takes about 10 s on a desktop computer. To get more accurate chemical shift values, one could use quantum chemical calculations at the expense of calculation time [31,32]. To make NMR parameters even more selective, one could include additional NMR parameters, in particularly the connectivity information retrieved from 2D ^1^H-^1^H TOCSY [23], 2D ^13^C-^1^H HSQC-TOCSY [33] or 2D ^13^C-^1^H HMBC [24]. Metabolites with high quaternary carbon content may require direct carbon detection experiments such as 1D ^13^C NMR and their comparison with 1D ^13^C NMR predictions. Successful implementation of the workflow requires detection of a metabolite of interest by both MS and NMR. Therefore, a metabolite should have at least low micromolar concentration to be detected in NMR experiments. Moreover, it should be ionized in a mass spectrometer. Signal overlaps are another challenge for the success of the approach, here we showed that the most straightforward way to resolve this challenge in real mixtures is to perform partial or complete LC-fractionation before NMR analysis. As we have shown with examples, our NMR/MS^2^ approach is designed to be able to work in all three conditions, switching from unfractionated to partially fractionated to completely fractionated sample depending on the experimental conditions does not significantly affect the protocol. To the best of our knowledge, this is the first study developed to facilitate the identification of unknown metabolites by combining NMR and MS/MS predictions, which supports previous findings that in addition to HRMS, NMR is required for de-novo identification of plant metabolites [34]. We used this approach to identify a type of glucosinolate, glucoraphanin in *A. thaliana*. Glucosinolates are biologically active secondary metabolites that occur in Brassicaceae species [35] such as food crops, broccoli and cauliflower. These metabolites influence plant-insect interactions, and glucoraphanin has been shown to be an important chemical for the insect pest resistance in *Arabidopsis* [36]. Glucoraphanin in vegetables within Brassicaceae is associated with a lower risk of lung and colorectal cancer [37]. Continued application of the NMR/MS^2^ approach is ongoing on plant derived metabolites. The unknown/uncatalogued metabolites identified will be added to metabolomics databases for database expansion and dereplication [38]. The NMR/MS^2^ approach is applicable to a wide range of samples from plant extracts, to, microbes, soils, dissolved organic matter, food extracts, biofuels and biomedical samples.

## 4. Materials and Methods

The model mixture was prepared by dissolving thymidine, proline, phenylalanine, pantothenate, nicotinate, methionine, isoleucine, glutamine, leucine and valine in 5 µL D_2_O, 45 µL H_2_O and 950 µL acetonitrile. The final concentration of each metabolite was 100 µM. 100 µL of this material was injected to LC-MS. NMR sample of the three-compound mixture was prepared by dissolving glutamine, valine and methionine in 180 µL D_2_O, the final concentration of each metabolite was 1 mM. *A. thaliana* metabolite extract was prepared as described previously [39]. The plant extract was dissolved in 200 µL D_2_O with 0.5 mM DSS, 180 µL of this sample was used for NMR. 5 µL of this sample was diluted with 45 µL H_2_O and 950 µL acetonitrile, 100 µL of this was injected to LC-MS.

2D ^13^C-^1^H HSQC spectra [22] were collected using a Varian (VNMRS) 600 MHz solution state NMR spectrometer equipped with a Varian z-gradient triple resonance HCN cold probe. The number of scans per t_1_ increment were 16 for the three-compound mixture, 64 for unfractionated *Arabidopsis* extract, 256 for fractionated *Arabidopsis* extract, and 128 for glucoraphanin standard (Cayman Chemicals, Ann Arbor, MI, USA). 2D ^1^H-^1^H TOCSY spectra for the unfractionated *Arabidopsis* extract and fractionated *Arabidopsis* extract were collected by 32 and 256 scans per t_1_ increment. TOCSY mixing time was 90 ms. The 2D ^13^C-^1^H HSQC, 2D ^1^H-^1^H TOCSY [23] and 2D ^13^C-^1^H HMBC [24] spectra of the ten metabolites were downloaded from BMRB database [8]. All data were zero-filled, Fourier transformed, phase and baseline corrected using NMRPipe [40].

Mass spectrometry studies were conducted using an HPLC system (1200 series, Agilent Technologies, Santa Clara, CA, USA) coupled to an Agilent 6538 UHD ESI Q-TOF instrument with a mass resolution of 40K. The instrument was externally calibrated with Agilent low-concentration tuning mix (part no. G1969-85000) before sample analysis, achieving a mass accuracy of ±5 ppm. LC-MS and collision induced dissociation LC-MS/MS data collection was performed together in auto MS/MS mode with a fixed collision energy of 30 V in both positive and negative ionization modes. Samples were separated using a normal-phase approach with a Phenomenex Luna NH_2_ column (150 mm × 2 mm i.d., 3 µm particle size). A gradient method was used for separation. The composition of the solvents was A = 10 mM NH_4_Ac plus 10 mM NH_4_OH in 95% water and 5% acetonitrile (mobile phase A) and solvent B = 95% acetonitrile and 5% water (mobile phase B). A 0.1 mL/min flow rate was used for the mobile phase flow, starting with 100%B to 0% B over 60 min, holding at 0% B for 5 min., increasing the flow rate to 0.2 mL/min and re-equilibration with 100% B for 54 min., then returning the flow to 0.1 mL/min for an additional 10 min. The settings for the mass spectrometer were as follows: capillary voltage, 4000 V (positive ion mode) and −4000 V (negative ion mode); drying gas flow (N_2_), 5 L/min; drying gas temperature, 330 °C; and nebulizer gas (N_2_), 30 psig. The raw LC-MS data was converted to mzdata.xml format by using MassHunter Qualitative Analysis (Agilent Technologies) and analyzed by mzMine 2 [41]. LC-fractionation of the plant extract was performed by an Agilent HPLC system (1200 series) coupled to an Agilent fraction collector (1100 series). The LC column, elution gradient, flow rates, solvents and injection volume were the same as the LC-MS experiments above. The fractions were collected in a 96-well deep plate (96DeepNunc31mm) with 1-min retention time intervals.

Candidate structures for the chemical formulas were extracted from ChemSpider database [21] by using ‘advanced’ and ‘intrinsic properties’ search option. 2D ^13^C-^1^H HSQC spectra for the candidate structures were predicted using MestReNova software (Santiago de Compostela, Spain). HSQC spectra of the predicted structures were compared with experimental HSQC spectrum and ranked by using a custom developed MATLAB algorithm. The ranking took into account chemical shift differences between experimental and predicted chemical shifts, and the matching ratio. The chemical shift differences were calculated by using a distance matrix as previously described [19,20]. The weights assigned for ^1^H and ^13^C chemical shifts were 10 and 1, respectively. The matching ratio is defined as the ratio of the matched peaks to the total number of peaks. For example, if a certain metabolite has 6 cross-peaks based on HSQC prediction, and 5 of them have corresponding matched peaks in the experimental HSQC spectrum, the matching ratio for this metabolite is 0.83. Sorting the metabolite candidates by minimum chemical shift difference and maximum matching ratio provided the quantitative ranking. Running the algorithm takes less than one second on a desktop computer. Generation of the MS/MS predictions for ChemSpider candidates and their comparisons with the experimental MS/MS spectrum was performed on MetFrag web server [15] using 30 ppm MS^2^ mass accuracy.

## Figures and Tables

**Table 1:** MS^2^ results for ten commonly known metabolites.

Metabolite	*m*/*z* ^a^	ppm ^b^	Formula	Size ^c^	Score ^d^	Rank ^e^
Phenylalanine	166.0859	2	C_9_H_11_NO_2_	1881	1.0000	1
Valine	118.0850	10	C_5_H_11_NO_2_	453	0.9240	6
Nicotinate	124.0392	0	C_6_H_5_NO_2_	100	0.8900	7
Pantothenate	220.1174	2	C_9_H_17_NO_5_	563	0.8963	8
Glutamine	147.0746	12	C_5_H_10_N_2_O_3_	283	0.8404	10
Methionine	150.0575	5	C_5_H_11_NO_2_S	212	0.8952	13
Isoleucine	132.1016	2	C_6_H_13_NO_2_	962	0.8723	22
Proline	116.0703	2	C_5_H_9_NO_2_	528	0.8515	26
Thymidine	243.0971	1	C_10_H_14_N_2_O_5_	948	0.9110	40
Leucine	132.1018	0	C_6_H_13_NO_2_	962	0.7002	148

^a^ Experimentally detected *m*/*z* ratio of [M + H]^+^; ^b^ *m*/*z* ratio difference (in units of ppm) between experimentally detected and theoretical *m*/*z* ratio of a given ion; ^c^ Number of structures for a given molecular formula (obtained with ChemSpider); ^d^ Matching score between experimental and predicted MS^2^ of a given metabolite (obtained with MetFrag), where 1.0 represents the best score. The score goes toward 0 as the agreement between experimental and predicted MS^2^ decreases; ^e^ Rank-ordered agreement between experimental and predicted MS^2^ of a given metabolite (obtained with MetFrag).

**Table 2:** NMR results for ten commonly known metabolites.

Metabolite	Size ^a^	^1^H ^b^	^13^C ^c^	Rank ^d^	Rank ^e^
Phenylalanine	1881	0.1224	2.8505	2	2
Valine	453	0.0773	0.6653	1	1
Nicotinate	100	0.1187	3.1317	1	1
Pantothenate	563	0.1320	0.8340	4	16
Glutamine	283	0.0852	1.4473	1	1
Methionine	212	0.2094	0.9670	1	1
Isoleucine	962	0.0923	2.2827	1	1
Proline	528	0.3077	1.0908	2	2
Thymidine	948	0.1959	2.0899	2	2
Leucine	962	0.1220	1.5720	1	3

^a^ Number of structures for a given molecular formula (obtained with ChemSpider); ^b^ Average ^1^H chemical shift difference (in units of ppm) between predicted and experimental chemical shifts for a given metabolite; ^c^ Average ^13^C chemical shift difference (in units of ppm) between predicted and experimental chemical shifts for a given metabolite; ^d^ Rank-ordered agreement between predicted and experimental chemical shifts of a given metabolite when the metabolite is the only unknown in the NMR spectrum; ^e^ Rank-ordered agreement between predicted and experimental chemical shifts of a given metabolite when the metabolite is one of the ten unknowns in the NMR spectrum.

## Supplementary Materials

The following are available online at www.mdpi.com/2218-1989/8/1/8/s1, Figures S1 and S2: chemical structures of the molecules, whose NMR predictions matched best to the experimental NMR spectra, Figure S3: total ion chromatogram of Arabidopsis metabolite extract, Figure S4: the MS/MS peak differentiating MS/MS prediction of glucoraphanin from the other isomers, Figures S5 and S6: overlay of the experimental 2D ^13^C-^1^H HSQC spectra of glucoraphanin, unfractionated and fractionated *Arabidopsis* extracts, Table S1: the list of precursor ions that were selected for fragmentation.

## References

[1] Nicholson J.K., Holmes E., Kinross J.M., Darzi A.W., Takats Z., Lindon J.C. (2012). Metabolic phenotyping in clinical and surgical environments. Nature.

[2] Bundy J.G., Davey M.P., Viant M.R. (2009). Environmental metabolomics: A critical review and future perspectives. Metabolomics.

[3] Martien J.I., Amador-Noguez D. (2017). Recent applications of metabolomics to advance microbial biofuel production. Curr. Opin. Biotechnol..

[4] Scalbert A., Brennan L., Manach C., Andres-Lacueva C., Dragsted L.O., Draper J., Rappaport S.M., van der Hooft J.J., Wishart D.S. (2014). The food metabolome: A window over dietary exposure. Am. J. Clin. Nutr..

[5] Ellis D.I., Goodacre R. (2012). Metabolomics-assisted synthetic biology. Curr. Opin. Biotechnol..

[6] Markley J.L., Brüschweiler R., Edison A.S., Eghbalnia H.R., Powers R., Raftery D., Wishart D.S. (2017). The future of NMR-based metabolomics. Curr. Opin. Biotechnol..

[7] Lu W., Su X., Klein M.S., Lewis I.A., Fiehn O., Rabinowitz J.D. (2017). Metabolite measurement: Pitfalls to avoid and practices to follow. Annu. Rev. Biochem..

[8] Ulrich E.L., Akutsu H., Doreleijers J.F., Harano Y., Ioannidis Y.E., Lin J., Livny M., Mading S., Maziuk D., Miller Z. (2008). BioMagResBank. Nucleic Acids Res..

[9] Wishart D.S., Jewison T., Guo A.C., Wilson M., Knox C., Liu Y., Djoumbou Y., Mandal R., Aziat F., Dong E. (2013). HMDB 3.0—The human metabolome database in 2013. Nucleic Acids Res..

[10] Zhu Z.J., Schultz A.W., Wang J., Johnson C.H., Yannone S.M., Patti G.J., Siuzdak G. (2013). Liquid chromatography quadrupole time-of-flight mass spectrometry characterization of metabolites guided by the METLIN database. Nat. Protocols.

[11] Bingol K., Li D.W., Zhang B., Brüschweiler R. (2016). Comprehensive metabolite identification strategy using multiple two-dimensional nmr spectra of a complex mixture implemented in the COLMARm web server. Anal. Chem..

[12] Koehn F.E., Carter G.T. (2005). The evolving role of natural products in drug discovery. Nat. Rev. Drug Discov..

[13] Wang Y., Kora G., Bowen B.P., Pan C. (2014). MIDAS: A database-searching algorithm for metabolite identification in metabolomics. Anal. Chem..

[14] Duhrkop K., Shen H., Meusel M., Rousu J., Bocker S. (2015). Searching molecular structure databases with tandem mass spectra using CSI:FingerID. Proc. Natl. Acad. Sci. USA.

[15] Ruttkies C., Schymanski E.L., Wolf S., Hollender J., Neumann S. (2016). Metfrag relaunched: Incorporating strategies beyond in silico fragmentation. J. Cheminform..

[16] Bingol K., Zhang F., Bruschweiler-Li L., Brüschweiler R. (2012). Carbon backbone topology of the metabolome of a cell. J. Am. Chem. Soc..

[17] Clendinen C.S., Pasquel C., Ajredini R., Edison A.S. (2015). ^13^C NMR metabolomics: Inadequate network analysis. Anal. Chem..

[18] Komatsu T., Ohishi R., Shino A., Kikuchi J. (2016). Structure and metabolic-flow analysis of molecular complexity in a ^13^C-labeled tree by 2D and 3D NMR. Angew. Chem..

[19] Bingol K., Bruschweiler-Li L., Yu C., Somogyi A., Zhang F., Brüschweiler R. (2015). Metabolomics beyond spectroscopic databases: A combined MS/NMR strategy for the rapid identification of new metabolites in complex mixtures. Anal. Chem..

[20] Wang C., He L., Li D.W., Bruschweiler-Li L., Marshall A.G., Brüschweiler R. (2017). Accurate identification of unknown and known metabolic mixture components by combining 3D NMR with fourier transform ion cyclotron resonance tandem mass spectrometry. J. Proteome Res..

[21] Pence H.E., Williams A. (2010). ChemSpider: An online chemical information resource. J. Chem. Educ..

[22] Bodenhausen G., Ruben D.J. (1980). Natural abundance N^15^ nmr by enhanced heteronuclear spectroscopy. Chem. Phys. Lett..

[23] Braunschweiler L., Ernst R.R. (1983). Coherence transfer by isotropic mixing—Application to proton correlation spectroscopy. J. Magn. Reson..

[24] Bax A., Summers M.F. (1986). H-1 and C-13 assignments from sensitivity-enhanced detection of heteronuclear multiple-bond connectivity by 2D multiple quantum NMR. J. Am. Chem. Soc..

[25] Bingol K., Li D.W., Bruschweiler-Li L., Cabrera O.A., Megraw T., Zhang F., Brüschweiler R. (2015). Unified and isomer-specific NMR metabolomics database for the accurate analysis of ^13^C-^1^H HSQC spectra. ACS Chem. Biol..

[26] Kind T., Fiehn O. (2006). Metabolomic database annotations via query of elemental compositions: Mass accuracy is insufficient even at less than 1 ppm. BMC Bioinform..

[27] Benecke C., Grund R., Hohberger R., Kerber A., Laue R., Wieland T. (1995). Molgen^+^, a generator of connectivity isomers and stereoisomers for molecular-structure elucidation. Anal. Chim. Acta.

[28] Kanehisa M., Goto S. (2000). KEGG: Kyoto encyclopedia of genes and genomes. Nucleic Acids Res..

[29] Allen F., Greiner R., Wishart D. (2015). Competitive fragmentation modeling of esi-ms/ms spectra for putative metabolite identification. Metabolomics.

[30] Metz T.O., Baker E.S., Schymanski E.L., Renslow R.S., Thomas D.G., Causon T.J., Webb I.K., Hann S., Smith R.D., Teeguarden J.G. (2017). Integrating ion mobility spectrometry into mass spectrometry-based exposome measurements: What can it add and how far can it go?. Bioanalysis.

[31] Chikayama E., Shimbo Y., Komatsu K., Kikuchi J. (2016). The effect of molecular conformation on the accuracy of theoretical ^1^H and ^13^C chemical shifts calculated by ab initio methods for metabolic mixture analysis. J. Phys. Chem. B.

[32] Hoffmann F., Li D.W., Sebastiani D., Brüschweiler R. (2017). Improved quantum chemical nmr chemical shift prediction of metabolites in aqueous solution toward the validation of unknowns. J. Phys. Chem. A.

[33] Lerner L., Bax A. (1986). Sensitivity-enhanced two-dimensional heteronuclear relayed coherence transfer nmr-spectroscopy. J. Magn. Reson..

[34] Tolstikov V., Costisella B., Weckwerth W., Zhang B., Fiehn O. Accurate mass QTOF and MSn Ion trap measurements require additional NMR data for plant metabolites de-novo identification. Proceedings of the 50th ASMS Conference on Mass Spectrometry and Allied Topics.

[35] Kliebenstein D.J., Kroymann J., Brown P., Figuth A., Pedersen D., Gershenzon J., Mitchell-Olds T. (2001). Genetic control of natural variation in arabidopsis glucosinolate accumulation. Plant Physiol..

[36] Beekwilder J., van Leeuwen W., van Dam N.M., Bertossi M., Grandi V., Mizzi L., Soloviev M., Szabados L., Molthoff J.W., Schipper B. (2008). The impact of the absence of aliphatic glucosinolates on insect herbivory in arabidopsis. PLoS ONE.

[37] Higdon J.V., Delage B., Williams D.E., Dashwood R.H. (2007). Cruciferous vegetables and human cancer risk: Epidemiologic evidence and mechanistic basis. Pharmacol. Res..

[38] Bingol K., Brüschweiler R. (2017). Knowns and unknowns in metabolomics identified by multidimensional nmr and hybrid ms/nmr methods. Curr. Opin. Biotechnol..

[39] Walker L.R., Hoyt D.W., Walker S.M., Ward J.K., Nicora C.D., Bingol K. (2016). Unambiguous metabolite identification in high-throughput metabolomics by hybrid 1D ^1^H NMR/ESI MS^1^ approach. Magn. Reson. Chem..

[40] Delaglio F., Grzesiek S., Vuister G.W., Zhu G., Pfeifer J., Bax A. (1995). Nmrpipe: A multidimensional spectral processing system based on unix pipes. J. Biomol. NMR.

[41] Pluskal T., Castillo S., Villar-Briones A., Oresic M. (2010). Mzmine 2: Modular framework for processing, visualizing, and analyzing mass spectrometry-based molecular profile data. BMC Bioinform..

