# Supplementary material for: Structure Elucidation of Unknown Metabolites in Metabolomics by Combined NMR and MS/MS Prediction
Source: Metabolites. 2018 Jan 17;8(1):8. doi: 10.3390/metabo8010008 (PMC5875998; doi:10.3390/metabo8010008)
Supplement: Supplementary file 1 [file metabolites-08-00008-s001.pdf]

**$C_5H_9NO_2$**<br>5-Hydroxy-2-piperidinone<br>NMR rank: 1<br>MS^2 rank: 167	**$C_5H_9NO_2$**<br>Proline<br>NMR rank: 2<br>MS^2 rank: 26
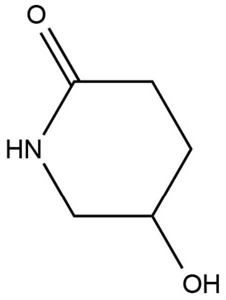	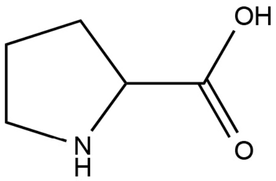

Predicted HSQC (5-Hydroxy-2-piperidinone)

^1H (ppm)	^{13}C (ppm)
1.69	29.69
2.09	29.69
2.17	30.63
2.51	30.63
3.22	46.12
3.57	46.12
4.12	66.37

Predicted HSQC (Proline)

^1H (ppm)	^{13}C (ppm)
1.75	23.93
1.84	31.27
2.04	31.27
2.87	46.72
3.04	46.72
3.85	63.51

Figure S1. Chemical structure of the best and second best match when HSQC predictions of all $C_5H_9NO_2$ isomers were queried against experimental HSQC of proline. HSQC prediction of 5-hydroxy-2-piperidinone was the best match (false positive) and proline was the second best match. However, when MS^2 predictions of all $C_5H_9NO_2$ isomers were queried against experimental MS^2 of proline, 5-hydroxy-2-piperidinone was the 167th match, much higher than proline prediction (26th match). Therefore, combining NMR with MS^2 helped discrimination of the false positive.

$C_6H_{13}NO_2$<br>Isoleucine<br>NMR rank: 1	$C_6H_{13}NO_2$<br>3-methylaminopropyl acetate<br>NMR rank: 2	$C_6H_{13}NO_2$<br>Leucine<br>NMR rank: 3
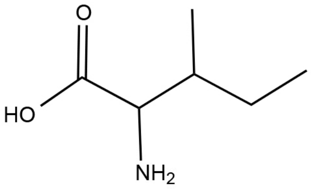	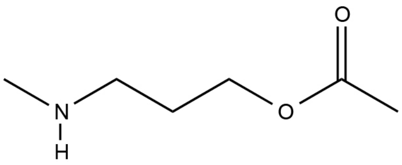	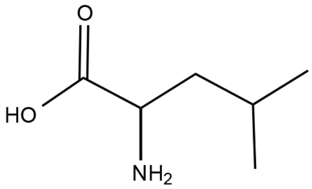
**Predicted HSQC**	**Predicted HSQC**	**Predicted HSQC**
^{1}H (ppm) ^{13}C (ppm)	^{1}H (ppm) ^{13}C (ppm)	^{1}H (ppm) ^{13}C (ppm)
0.99 11.60	2.02 20.55	0.95 22.46
1.10 14.98	1.88 29.61	1.64 24.55
1.31 24.17	2.42 33.39	1.60 42.20
1.78 24.17	2.93 49.62	2.56 42.20
1.65 36.67	4.15 62.95	3.27 53.60
3.06 61.17		

Figure S2. Chemical structure of the best, second and third best match when HSQC predictions of all $C_6H_{13}NO_2$ isomers were queried against deconvoluted experimental HSQCs of thymidine, proline, phenylalanine, pantothenate, nicotinate, methionine, isoleucine, glutamine, leucine and valine. Isoleucine HSQC prediction found its experimental HSQC as the best match, 3-methylaminopropyl acetate HSQC prediction found experimental HSQC of proline as the second best match (false positive). Finally, leucine HSQC prediction found experimental HSQC of leucine as the third best match.

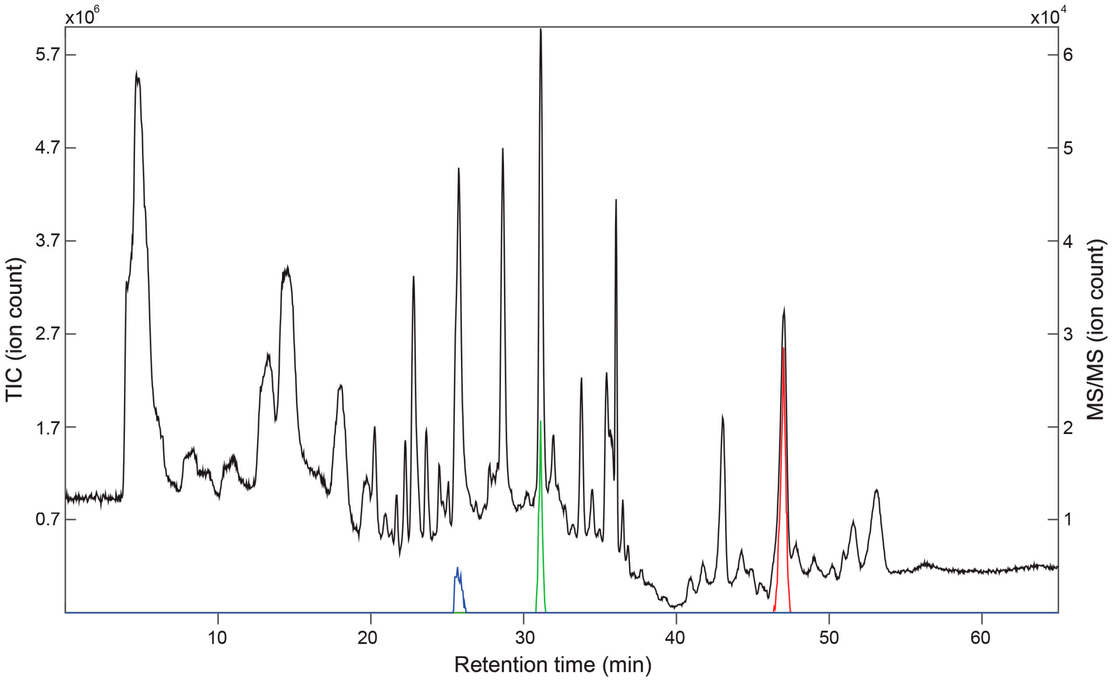

Figure S3. Total ion chromatogram (black) of *A. thaliana* metabolite extract. Overlaid LC-MS/MS chromatogram of three compounds selected based on precursor and daughter ions; blue (precursor *m*/*z*: 367.0976, daughter *m*/*z*: 115.0171, retention time: 25.8 min), green (precursor *m*/*z*: 341.1032, daughter *m*/*z*: 59.0114, retention time: 31.1 min) and red (precursor *m*/*z*: 436.0310, daughter *m*/*z*: 96.9567, retention time: 47.0 min). The red chromatogram belongs to glucoraphanin (see Figure 3).

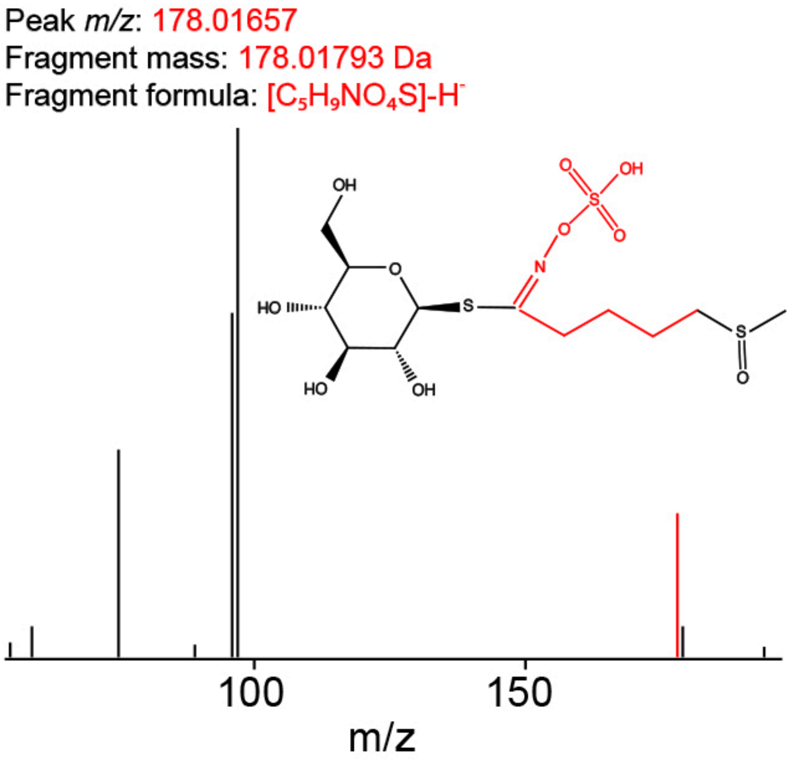

Figure S4. The MS/MS peak (red) differentiating MS/MS prediction of structure **c** from structure **a** and **b** in Figure 3. The peak experimentally appeared at ~*m*/*z* 178.0166. It matched to the theoretical mass of the fragment formula of $[C_5H_9NO_4S]$-H^- including the SO_4 group and part of the aliphatic chain of glucoraphanin (red).

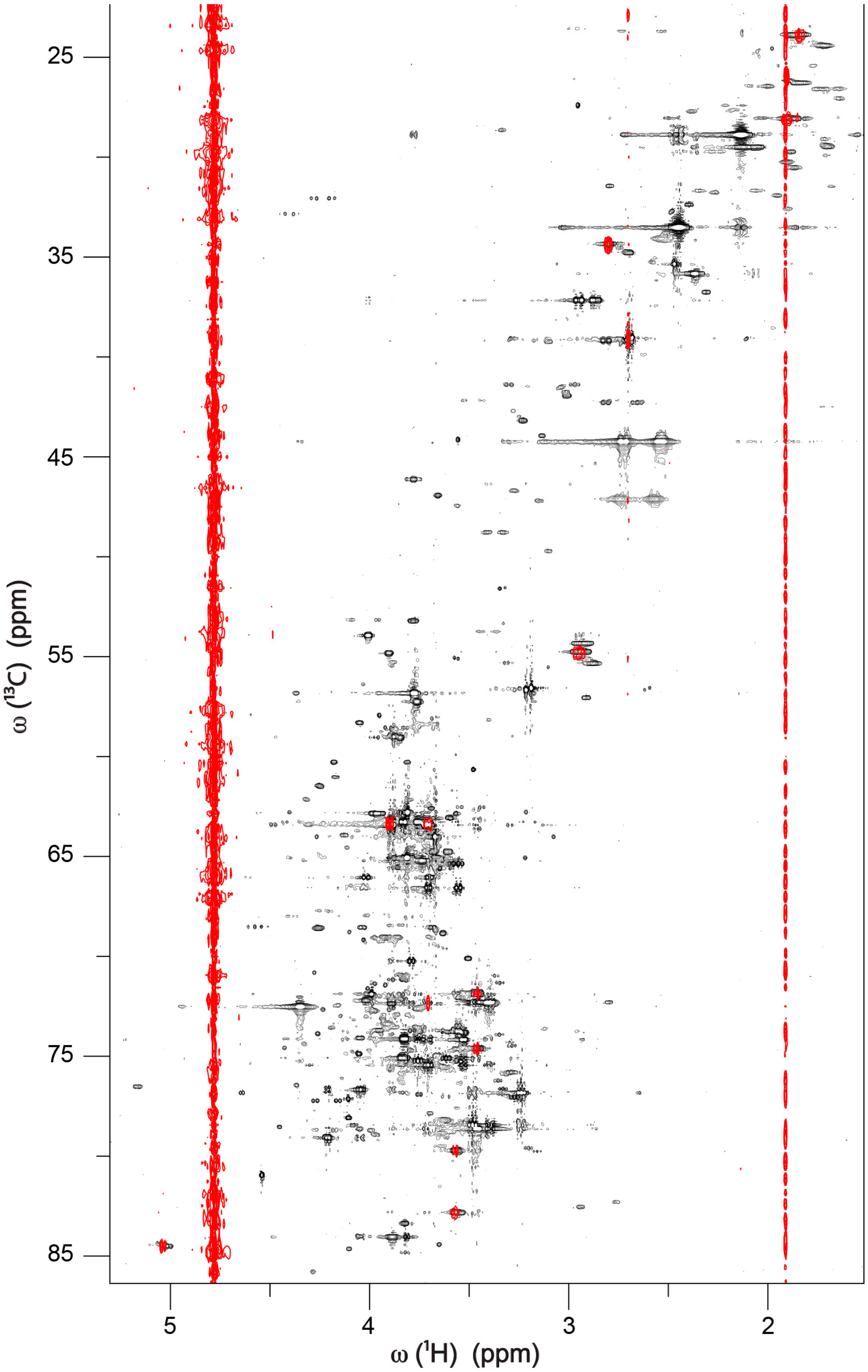

Figure S5. Overlay of the experimental 2D ^{13}C-^{1}H HSQC spectra of the unfractionated *A. thaliana* extract (black) and LC-fractionated *A. thaliana* extract at the retention time 47 min (red).

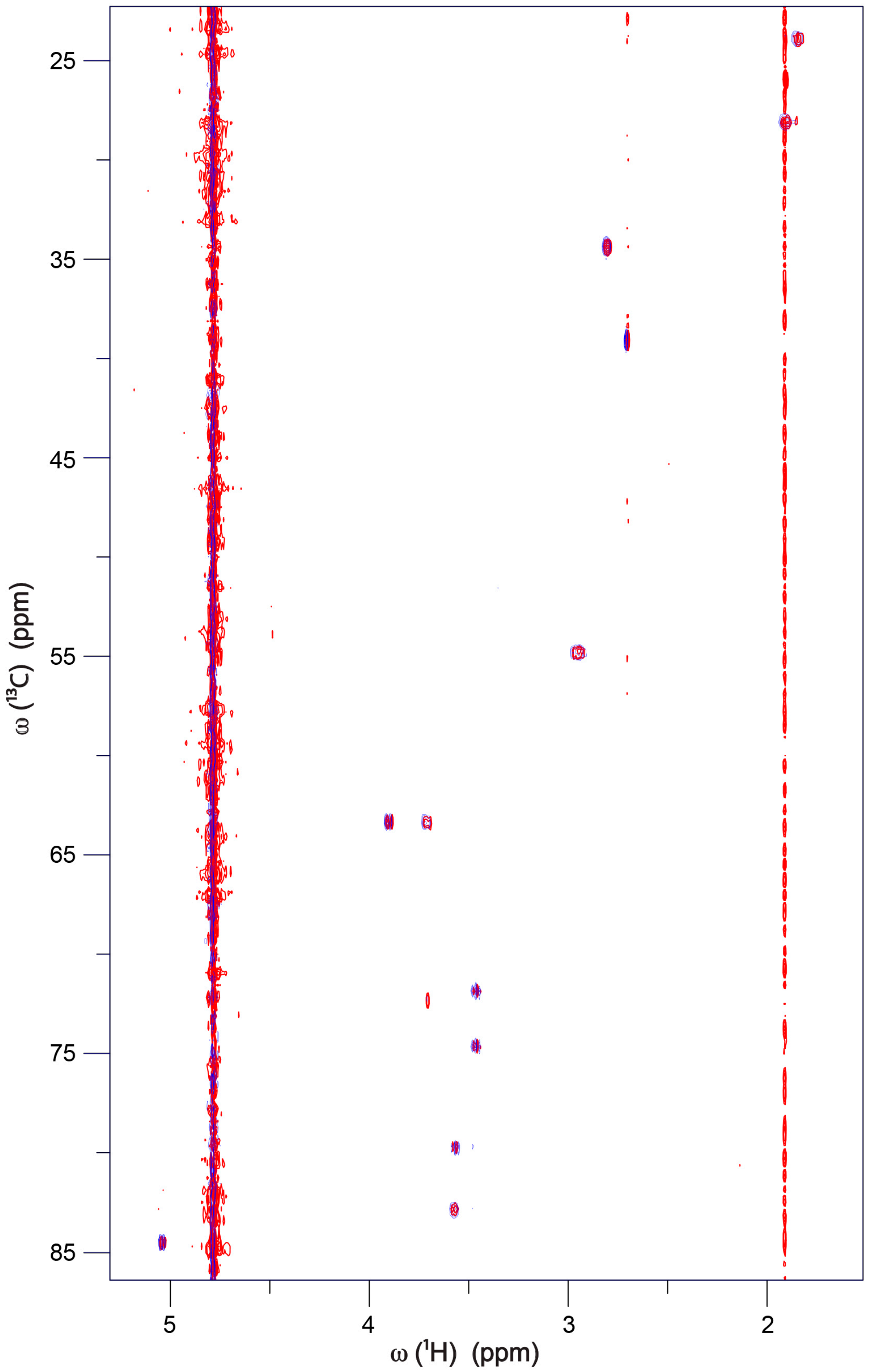

Figure S6. Overlay of the experimental 2D ^{13}C-^{1}H HSQC spectra of the LC-fractionated *A. thaliana* extract at the retention time 47 min (red) and glucoraphanin standard (blue).

Table S1. The list of precursor ions that were selected for fragmentation in this study

metabolite	precursor *m/z*	retention time (min)	collision energy (V)	ion mode	daughter *m/z* list[a]
Thymidine	243.0967	12.2	30	+	127.0499
					110.0235
					69.0336
					84.0444
					109.0398
Proline	116.0701	30.8	30	+	70.0651
					71.0683
					68.0489
					57.0566
					70.0908
Phenylalanine	166.0857	31.9	30	+	103.0538
					120.0803
					77.0385
					79.0540
					93.0689
Pantothenate	220.1170	45.4	30	+	70.0292
					67.0543
					57.0703
					90.0548
					98.0231
Nicotinate	124.0390	47.1	30	+	78.0335
					80.0491
					53.0384
					79.0403
					52.0186
Methionine	150.0575	32.1	30	+	61.0108
					56.0496
					59.0727
					58.0653
					63.0063
Isoleucine	132.1015	29.0	30	+	69.0698
					58.0661
					71.0718
					57.0565
					70.0729
Glutamine	147.0746	35.3	30	+	84.0431
					56.0488
					85.0273
					105.0321

					56.4253
Leucine	132.1010	28.3	30	+	55.0540
					86.0963
					53.0380
					55.0173
					69.0691
Valine	118.0850	30.5	30	+	55.0540
					57.0569
					72.0798
					56.0496
					58.0642
Glucoraphanin	436.0310	47.0	30	-	96.9567
					95.9484
					74.9883
					98.9526
					178.0166

[a] Only the highest intensity (top five) daughter ions were included in the table.
